# The number of retrieved lymph nodes needed for accurate staging differs based on the presence of preoperative chemoradiation for rectal cancer

**DOI:** 10.1097/MD.0000000000004891

**Published:** 2016-09-23

**Authors:** Jeonghee Han, Gyoung Tae Noh, Shen Ann Yeo, Chinock Cheong, Min Soo Cho, Hyuk Hur, Byung Soh Min, Kang Young Lee, Nam Kyu Kim

**Affiliations:** Department of Surgery, Severance Hospital, Yonsei University College of Medicine, Seoul, South Korea.

**Keywords:** lymph node, preoperative chemoradiotherapy, rectal cancer, stage migration

## Abstract

The aim of this study is to investigate if retrieval of 12 lymph nodes (LNs) is sufficient to avoid stage migration as well as to evaluate the prognostic impact of insufficient LN retrieval in different treatment settings of rectal cancer, particularly in the case of preoperative chemoradiotherapy (pCRT).

The data of all patients with biopsy proven rectal adenocarcinoma who underwent curative surgery between January 2005 and December 2012 were analyzed. Univariate and multivariate analyses for oncologic outcomes were performed in LN metastasis or no LN metastasis (LN−) group. Subgroup analyses were performed according to whether a patient had received pCRT.

A total of 1825 patients were enrolled into the study. The maximal Chi-square method revealed the minimum number of harvested LNs required to be 12. Univariate and multivariate analyses found LNs ≥ 12 to be an independent prognostic factor for both overall survival (OS) (hazard ratio [HR] = 0.5, 95% confidence intervals [CIs]: 0.3–0.8; *P* = 0.002) and disease-free survival (DFS) (HR = 0.6, 95% CI: 0.4–0.7; *P* < 0.001) in the LN− group. In the LN− group, LNs ≥ 12 continued to be a significant prognostic factor both for OS and DFS in the subgroup of patients who did not undergo pCRT. However, in the subgroup of the LN− patients who underwent pCRT, LN ≥ 8 was significant for DFS and OS.

Retrieval of LNs ≥ 12 and LNs ≥ 8 should be achieved to obtain accurate staging and optimal treatment for the non-pCRT and pCRT groups in rectal cancer, respectively.

## 1 Introduction

Colorectal cancer is the 3rd most common cause of cancer death in the USA,^[1]^ and in Korea the incidence of colorectal cancer has rapidly increased by 5.2% over the past 10 years. Locally, colorectal cancer is the 2nd most common cancer in males and the 3rd most common in females.^[2]^ Furthermore, in Korea, mortality from colorectal cancer is 3rd most common in males and 2nd most common in females.^[2]^

The prognosis for colorectal cancer is primarily determined by the tumor–node–metastasis (TNM) stage of the disease. The currently most widely accepted staging system for colorectal cancer – the 7th American Joint Committee on Cancer staging system – is based on the number of metastatic lymph nodes (LNs) present as well as the pathologic T-stage.^[3]^ Based on many reports, positive LN metastases have been well established as one of the most significant risk factors affecting recurrence and survival in colorectal cancer.^[4,5]^

Accurate diagnosis of the extent of disease is essential in the treatment of any malignancy, and rectal cancer is of no exception. Insufficient retrieval of LNs may result in stage migration, subsequently leading to suboptimal treatment – which may explain survival discrepancies between patients in the same stage of disease. Specifically, the possibility of stage migration is increased when the insufficient number of LNs have been harvested after resection and where no metastatic LNs have been found. The importance of the number of harvested LNs on outcomes of colorectal cancer has been identified in population-based and clinical studies.^[6,7]^ Although the current staging system recommends adequate number of harvested LNs of 12 or more, it disregards the location of the tumor being in the colon or rectum and the potential effect of different treatment strategies. Recently, preoperative chemoradiotherapy (pCRT) has become the standard approach for locally advanced rectal cancer.^[8]^ Since pCRT potentially induces LN regression, the chance of harvesting an inadequate number of LNs following surgery may increase.^[9]^

Furthermore, the data supporting this recommendation is based primarily on studies of patients undergoing surgery for colon cancer.^[10]^ In patients with rectal cancer, studies have shown that the LN harvest may be less compared to colon cancer,^[11]^ and as such the extent to which this indicative value of 12 LNs should be extrapolated to rectal cancer is certainly questionable.

For rectal cancer, despite guidelines suggesting 12 LNs to be the minimum requirement for the accurate assessment of LN status, opinions on the optimal number of harvested nodes differ between studies and varies according to different treatment settings.^[12]^ The aim of this study is to investigate if 12 LNs are sufficient to avoid stage migration and to evaluate the prognostic impact of insufficient LN retrieval in different treatment settings of rectal cancer.

## 2 Materials and methods

### 2.1 Patient enrollment

**Figure 1:**
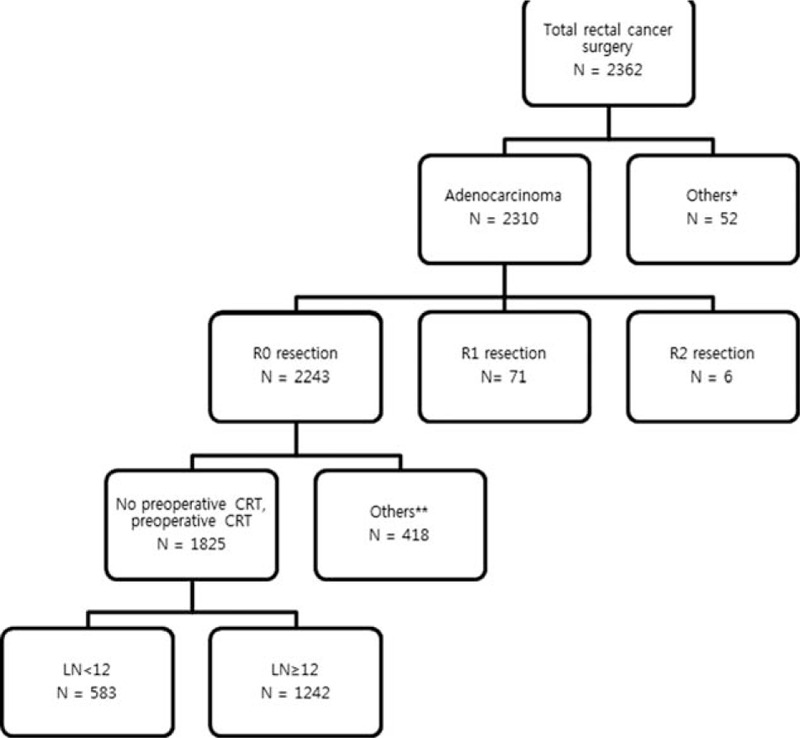
Overview of patient population in the study.

The medical records of the patients with biopsy proven rectal adenocarcinoma who underwent curative surgery between January 2005 and December 2012 were retrospectively reviewed. We excluded patients who underwent emergency operations, those with distant metastasis, lateral pelvic node dissection, and no available follow up data. A total of 1825 patients who met the enrollment criteria were divided into 2 groups with or without pCRT (Fig. 1).

### 2.2 Treatment and follow-up strategies

American Joint Committee on Cancer classification 7th edition^[3]^ was used for staging. Clinical stage was determined based on preoperative examination by chest radiography and/or chest computed tomography (CT) scan, abdominal and pelvic CT scan, transrectal ultrasonography, and/or pelvic magnetic resonance imaging (MRI). The pathologic examination was reported in standardized manner by qualified pathologists. Pathologic findings also described tumor differentiation, vascular invasion, lymphatic invasion, and resection margin in all specimens in addition to the T and N staging. Management of patients was discussed in multidisciplinary team meeting as necessary before commencement of treatment. Patients with clinically staged T3 or N1 disease received pCRT. In our institution, pCRT consists of 5-fluorouracil (5-FU)-based chemotherapy and pelvic irradiation (4500–5040 cGy) delivered in 25 fractions of 180 cGy/day over 5 weeks. Curative resection was performed 6 to 8 weeks after completion of pCRT and all patients received surgical resection based on the principles of total mesorectal excision (TME). Patients who underwent curative surgery were subjected to a standard follow-up program. Rectal examinations and serum carcinoembryonic antigen (CEA) measurements were carried out whenever the patient visited the outpatient clinic. Contrast-enhanced helical CT was performed every 6 months during the follow-up period, and MRI or positron emission tomography-CT was performed if needed.

### 2.3 Data analysis and statistical analyses

The primary outcome was disease-free survival (DFS) and overall survival (OS). DFS was defined as the time between date of surgery and date of recurrence. Recurrence included local, regional, and distant failures. Local and distant recurrences were confirmed radiologically or histologically by qualified radiologists and pathologists. OS was defined as the time between the date of surgery and the date of death or last follow-up. This study was approved by the institutional review board and informed consent was waived.

Statistical analysis was performed using SPSS version 20.0 (IBM corp., Armonk, NY) and R version 3.2.2 (The R Foundation for Statistical Computing Platform, Vienna, Austria) after patient-identifiers were removed from the dataset. Differences between categorical variables were compared using the χ^2^ test. The Wilcoxon rank sum test or *t* test was used for continuous variables by Shapiro–Wilk normality test. The Kaplan–Meier method was used to estimate OS and DFS with the log-rank test to compare factors. Multivariate Cox proportional hazard analysis was used to identify factors associated with DFS and OS.^[13]^ A *P* value of <0.05 was considered statistically significant.

## 3 Results

### 3.1 Patient clinical characteristics

A total of 1825 patients were enrolled in the study. There were 1159 (63.5%) men and 666 (36.5%) women, with a median age of 60 (range, 22–99 years). The minimum required harvested LN number was decided using maximal Chi-square method.^[14]^ The cut-off value of 12 LNs was determined by the maximal Chi-square method based on survival data (M = 3.2926, *P* = 0.02). Based on this number, the patients were divided into 2 groups, group A (<12 LNs) and group B (≥12 LNs). Clinical characteristics and pathological variables were compared accordingly. Patients in group A were more likely to be of older age (*P* < 0.001). Tumor location of group A was more likely to be located in lower rectum than group B (38.1% vs 26.3%, *P* < 0.001). More patients in group A were treated by pCRT than group B (41.2% vs 35.3%, *P* < 0.001). A lower concentration of preoperative CEA was found in group A (82.3% vs 70.7%, *P* < 0.001). The median follow-up was 52 months.

### 3.2 Pathologic characteristics with distribution of lymph nodes

The median number of LNs harvested was 15 (interquartile range [IQR], 10–22). In total, 1242 (68.1%) of patients had LNs ≥ 12 retrieved. The pathologic complete remission (pCR) rate after pCRT was significantly different between the groups (group A: 5.3% vs group B: 2.1%, *P* < 0.001). Lymphovascular invasion was less frequent in group A compared to group B (*P* < 0.001). There were 461 (79.1%) stage I/II patients in group A, but 747 (60.1%) stage I/II patients in group B (p < 0.001). Tumor grading showed no significant differences between the groups. The clinicopathologic characteristics are shown in Table 1.

**Table 1:**
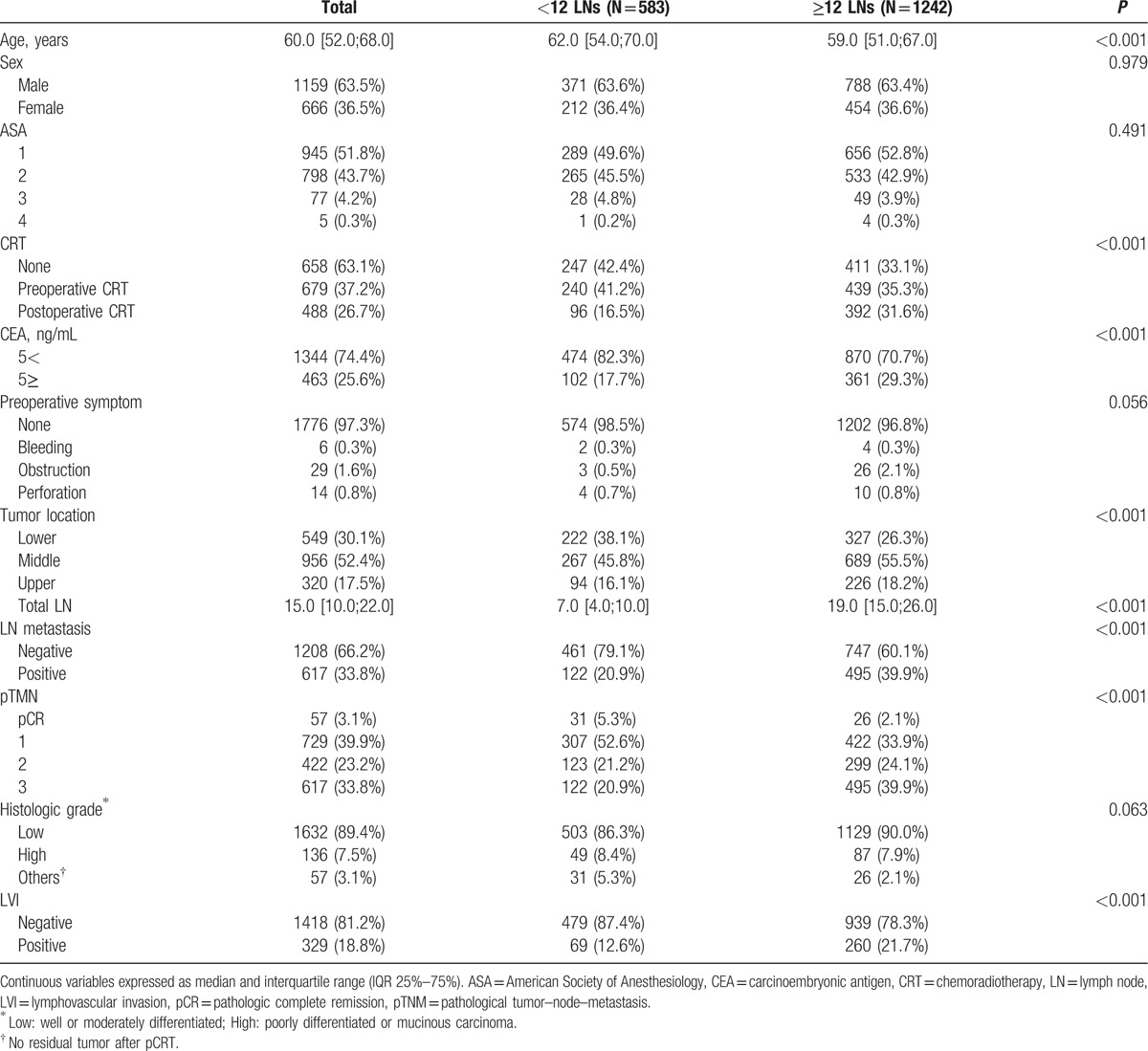
Clinicopathologic characteristics.

### 3.3 Survival in relation to harvested number of lymph nodes

**Figure 2:**
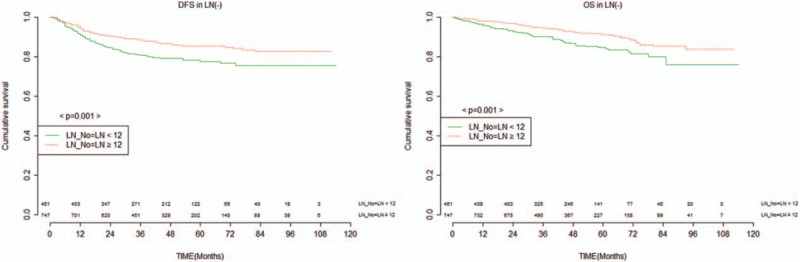
Disease-free survival and overall survival for 1208 patients with node negative.

Median DFS was 42 (range, 0–114) months in group A and 38 (range, 0–113) months in group B (*P* = 0.91). Median OS was 49 (range, 0–114) months in group A and 46 (range, 0–113) months in group B (*P* = 0.89). In the patients with metastatic LNs (n = 617), no significant difference in DFS (58% vs 56%, *P* = 0.383) and OS (73% vs 67%, *P* = 0.061) was observed in between group A and B. On the other hand, in patients without metastatic LN (n = 1208), a significant difference in DFS (80% vs 86%, *P* = 0.001) and OS (85% vs 92%, *P* = 0.001) was observed in between group A and B (Fig. 2).

**Figure 3:**
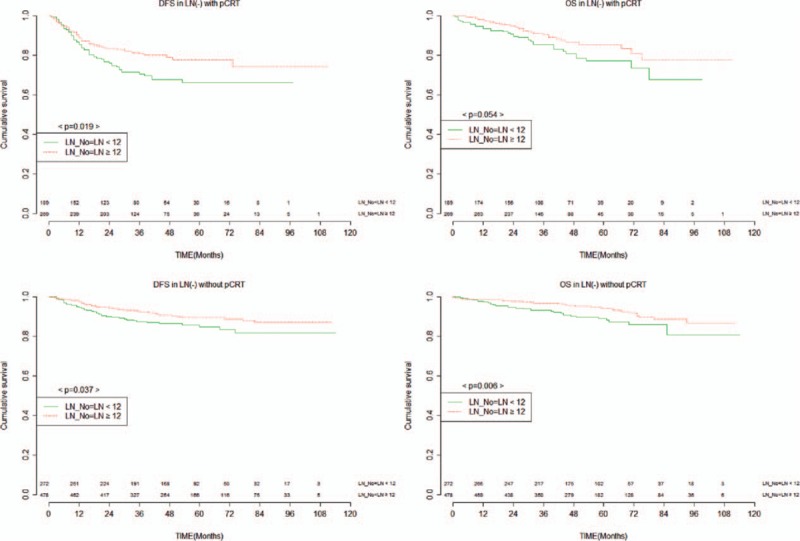
DFS and overall survival in patients with pCRT (n = 458) or not (n = 750) in node negative by 12 harvested nodes. DFS = disease-free survival, pCRT = preoperative chemoradiotherapy.

Patients without metastatic LNs were then further divided into those who did and did not undergo pCRT (Fig. 3). In the group without pCRT (n = 750), a significant difference in DFS (86% vs 91%, *P* = 0.037) and OS (89% vs 94%, *P* = 0.006) was observed in between group A and group B. In the pCRT group (n = 458), a significant difference in DFS (71% vs 80%, *P* = 0.019) was observed in between group A and group B. However, there was no significant difference in OS (81% vs 88%, *P* = 0.054).

### 3.4 Risk factor analysis in patients without metastatic lymph nodes

We performed uni- and multivariate analyses for the factors associated to DFS and OS in the patients without metastatic LNs (Tables 2, 3). On univariate analysis, LN ≥ 12, pathological TNM (pTNM) stage, pCRT, preoperative CEA, and tumor location were significant predictors of DFS. LN ≥ 12, age, preoperative CEA, pTNM stage, pCRT, tumor location, and preoperative symptoms were significant predictors of OS. On multivariate analysis, LNs ≥ 12 provided significant benefit in DFS compared with LNs < 12 (hazard ratio [HR] = 0.6, 95% confidence intervals [CIs]: 0.4–0.7; *P* *<* 0.001). Tumor location, pCRT, and pTNM stage were also significant independent predictors of DFS. We detected a significant difference in OS between groups with the number of LNs ≥ 12 and LNs < 12 on multivariate analysis (HR = 0.5, 95% CI: 0.3–0.8; *P* *=* 0.002). Age, pTNM stage, tumor location, and preoperative symptoms were significant independent predictors of OS.

**Table 2:**
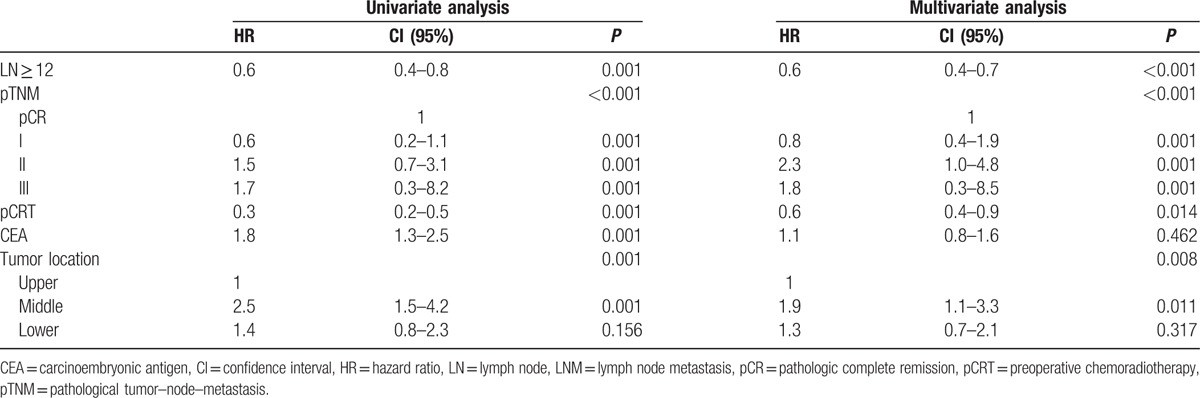
Cox regression hazard ratio for disease-free survival (LNM−).

**Table 3:**
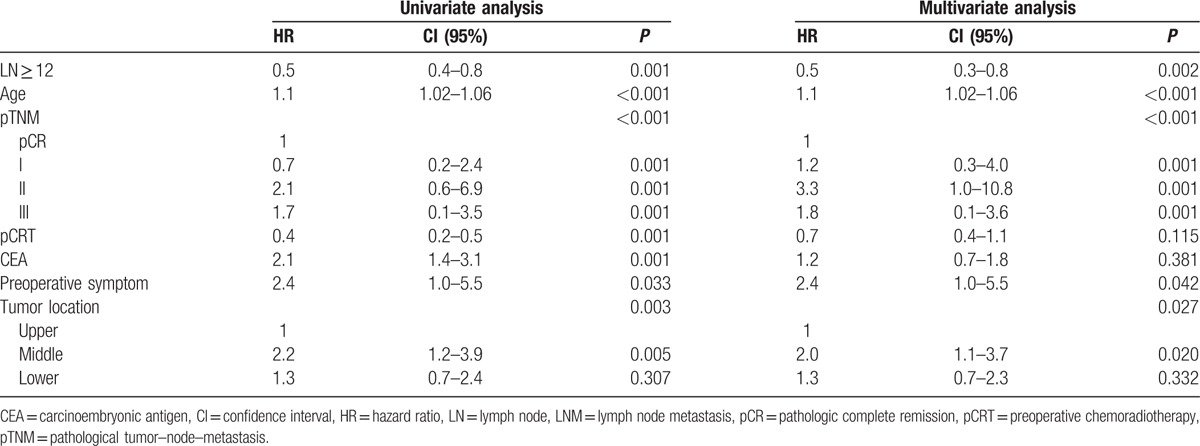
Cox regression hazard ratio for overall survival (LNM−).

In the patients with metastatic LNs, sex, lymphovascular invasion (LVI), preoperative CEA, pCRT, and pTNM stage were significant independent predictors of DFS. In addition, age, histologic grade, LVI, preoperative symptoms, and pTNM stage were significant independent predictors of OS. However, retrieval of 12 or more LNs provided no significant difference in both DFS and OS (Tables 4, 5).

**Table 4:**
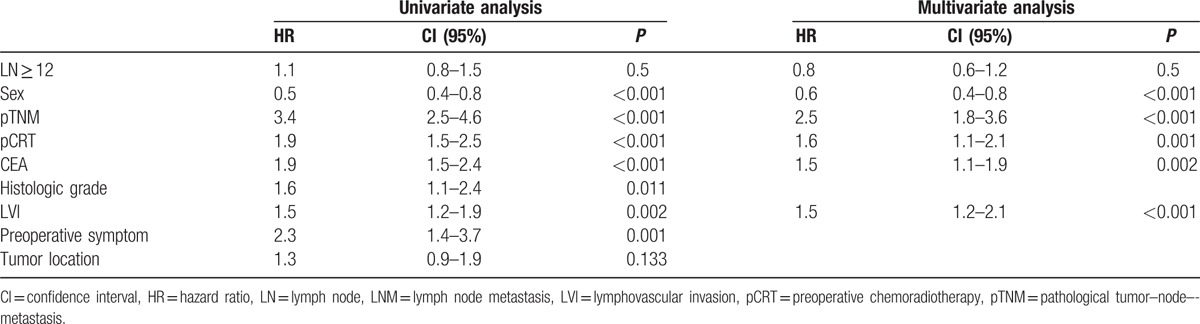
Cox regression hazard ratio for disease-free survival (LNM+).

**Table 5:**
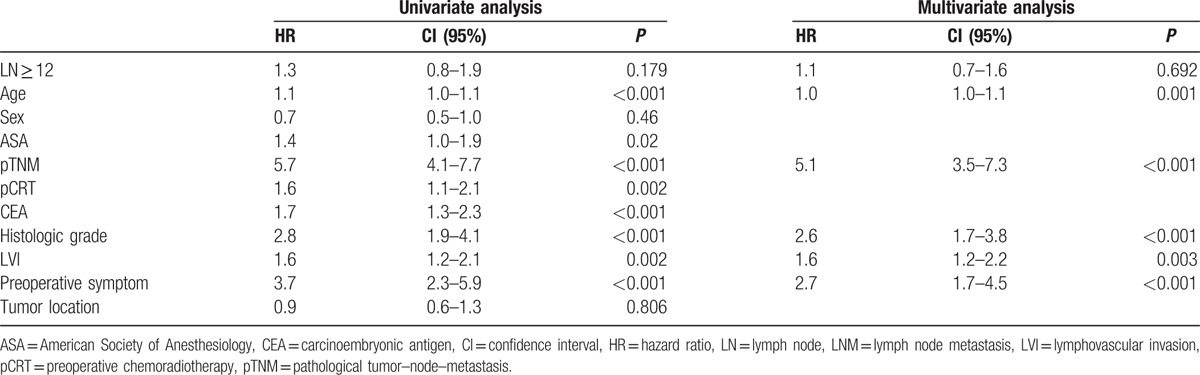
Cox regression hazard ratio for overall survival (LNM+).

### 3.5 Risk factor analysis in the patients without metastatic lymph node according to treatment settings

The patients without metastatic LNs were further divided into pCRT and no pCRT subgroups for further analyses. We performed uni- and multivariate analyses of the factors associated with DFS and OS in the patients without pCRT. In univariate analysis, LN ≥ 12, pTNM stage, LVI, preoperative CEA, and age were significant factors of DFS, and LN ≥ 12, age, preoperative CEA, pTNM stage, American Society of Anesthesiologists, and LVI were significant predictors of OS. In multivariate analysis, LNs ≥ 12 provided significant benefit in DFS compared with LNs < 12 (HR = 0.4, 95% CI: 0.2–0.7; *P* = 0.001). Age and pTNM stage were significant independent predictors of DFS. We detected significant independent difference in OS between the number of LNs ≥12 and LNs < 12 on multivariate analysis (HR = 0.4, 95% CI: 0.2–0.7; *P* *=* 0.002). Age, pTNM stage, and preoperative CEA were significant independent predictors of OS. In the patients with pCRT, LN ≥ 12 was a significant prognostic factor for DFS on multivariate analysis (HR = 0.6, 95% CI: 0.4–0.9; *P* = 0.021). However, it was not for OS (HR = 0.6, 95% CI: 0.4–1.1; *P* *=* 0.130).

### 3.6 Analysis for pCRT group without lymph node metastasis

**Figure 4:**
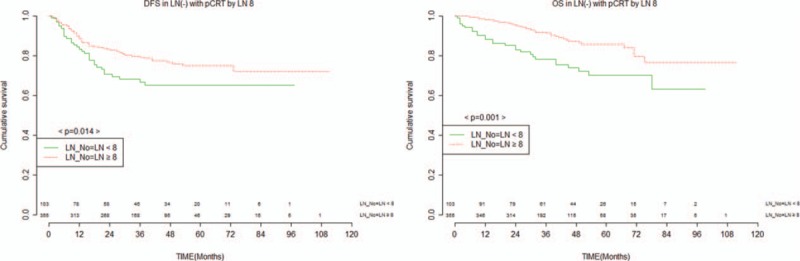
DFS and overall survival for 458 patients with pCRT and node negative by 8 harvested nodes. DFS = disease-free survival, pCRT = preoperative chemoradiotherapy.

Because LNs ≥ 12 was not a significant factor for OS in the pCRT group without LN metastasis, we suspected that the number of LN 12 might not be the optimal requirement in the setting of pCRT. As such, we performed further analysis for the pCRT group without LN metastasis to assess the minimum required harvested LN number using the maximal Chi-square method based on survival data.^[14]^ On the basis of this method, a cut-off value of 8 LN was determined (M = 3.4591, *P* = 0.015). A significant difference in DFS (69.9% vs 78.6%, *P* = 0.014) was observed between LNs ≥ 8 and LNs < 8. Furthermore, a significant difference in OS (73.8% vs 88.7%, *P* = 0.001) was observed between LNs ≥ 8 and LNs < 8 (Fig. 4). LN ≥ 8, pTNM stage, and histologic grade were significant factors for DFS and LNs ≥ 8, age, pTNM stage, histologic grade, and American Society of Anesthesiologists were significant predictors for OS on univariate analysis. On multivariate analysis, examination of LNs ≥ 8 provided a significant benefit in both DFS (HR = 0.6, 95% CI: 0.4–1.1; *P* = 0.042) and OS (HR = 0.5, 95% CI: 0.2–0.9; *P* = 0.002) compared to LNs < 8 (Tables 6, 7).

**Table 6:**
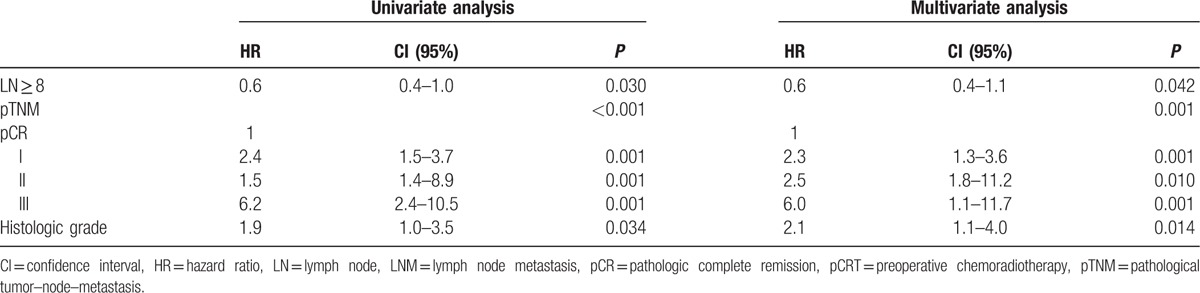
Cox regression hazard ratio for disease-free survival (LNM− with pCRT).

**Table 7:**
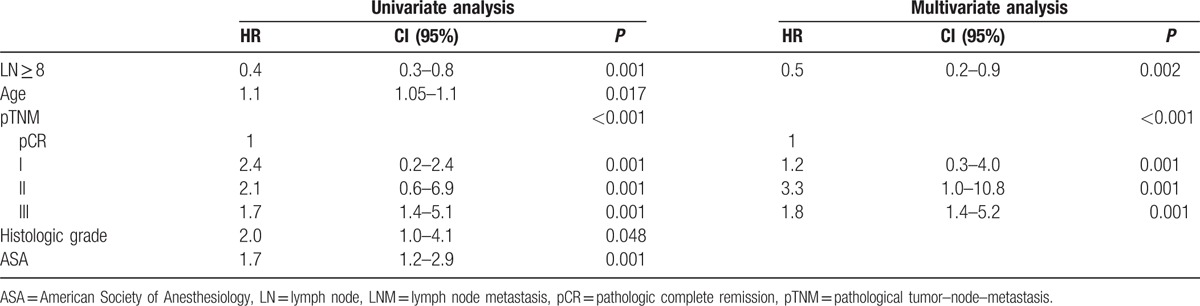
Cox regression hazard ratio for overall survival (LNM− with pCRT).

## 4 Discussion

Adequate LN dissection not only reduces the likelihood of local recurrence, but also enables the accurate staging of disease. Therefore, there is concern that retrieval of insufficient LNs may result in understaging of the disease, which consequently leads to suboptimal treatment. At this point in time, a minimum of 12 harvested LNs are recommended to accurately stage patients with both colon and rectal cancer.^[15,16]^ However, the studies supporting this minimum number of 12 LNs are derived mainly from studies on colon cancer.^[6,7]^ Recent studies have proposed that the LN yields in rectal specimens tend to be lower than those in colon specimens.^[11]^ Despite certain similarities in biology, the treatment paradigms of colon and rectal cancer are significantly different, and due to this both have differing recurrence rates and patterns.^[17]^ In rectal cancer, pCRT is employed in selected patients to improve locoregional control and functional outcomes,^[8,18–20]^ but it has also been shown to significantly reduce the number of LNs harvested compared to those treated without pCRT.^[21–26]^ Our study aims to assess if the harvesting of 12 LNs in rectal cancer is appropriate for accurate prognositication, taking into account the presence or absence of pCRT.

Surgical specimens need to be dissected by qualified pathologist with enough time to perform a thorough LN harvest if accurate staging is to be achieved. The small size of retrieved LNs in specimen is considered to be a limitation about standard methods for examining LNs. Rodriguez-Bigas et al^[27]^ reported that up to 70% of LNs with metastasis are <5 mm in diameter. Such LNs can more easily be missed if the pathologist applied a palpation technique for identifying LNs. In an effort to improve LN harvest, our institution routinely uses LN clearing solutions. Fat clearing solutions (xylene and alcohol) have been adopted in many centers to aid in LN retrieval, leading to an increase in their LN counts from 6.1 to 18.9,^[28]^ from 10.5 to 23.1,^[29]^ and from 18.1 to 76.4.^[30]^ The method with which LNs were harvested and analyzed in our study is thus standardized and rigorous.

In the patients with metastatic LNs (LN+), regardless of pCRT status, we found that the number of harvested LNs did not prognosticate DFS or OS. As such, our data suggest that for the patients with metastatic LNs, it may not be meaningful to require retrieval of more than 12 LNs, since LN positivity already such a significant prognostic factor for recurrence and survival.^[4,5]^

On the other hand, in patients without metastatic LNs (LN−), we have demonstrated that the number of harvested LNs was a significant prognostic factor, with an improvement in both DFS and OS in patients with more than 12 harvested LNs. In the subgroup of the LN− patients without pCRT, LNs ≥ 12 continued to be a significant prognostic factor both for OS and DFS. Thus, in this group of patients, a minimum of 12 LNs would be necessary to avoid understaging and stage migration.

However, in the subgroup of LN− patients with pCRT, LNs ≥ 12 was significant only for DFS, but not for OS. This result may be due to the effect of pCRT. Preoperative CRT influences LN status due to tumoricidal effects and significantly reduces the number of harvested LNs.^[9,24]^ The immune response and fibrosis of LNs after pCRT also contributes to the difficulty in identification of previously metastatic LNs in the pathologic specimens.^[22]^ Preoperative CRT may also decrease the size of nonmetastatic LNs by 1 to 2 mm^[31,32]^ and thus may decrease the likelihood of identification in the surgical specimen.^[33]^

The optimal number of examined LNs after pCRT still has not been fully elucidated. We attempted to investigate this further by performing analysis for the patients who underwent pCRT without LN metastasis. Using the maximal Chi-square method based on survival data (M = 3.4591, *P* = 0.015), we derived that the minimum number of LN required for examination that made a prognostic difference was 8. For the patient who underwent pCRT group with LN−, examination of LN ≥ 8 was a significant prognostic factor both for OS and DFS. Based on our data, an LN count of less than 8 in patients who have undergone pCRT and found to be LN− on pathology may be suboptimal and lead to understaging. Therefore, more than 8 examined LNs in this group of patients would be required to avoid stage migration.

Stage migration is an important factor to explain discrepancies of survival within the same stage. One of the most important reasons for stage migration in colorectal cancer is insufficient LN harvest. Given that the number of positive LNs identified is fundamentally related to the total number of harvested LNs, a proportion of patients classified as LN− are likely to have been understaged if the total number of LNs harvested was inadequate.^[34]^ Thus, evaluating a sufficient number of LNs will decrease the risk of missing a positive LN.

When patients with false-negative stage I/II are correctly rediagnosed as stage III, the prognosis of both the stage I/II and stage III patients will improve. This phenomenon – named after the famous American comedian – is known as the Will Rogers phenomenon.^[35]^ This is due to 2 factors. First, the prognosis of patients being rediagnosed is below average for the group (stage I/II). Removing these patients will, by definition, raise the average of the remaining patients group. Second, the prognosis of patients being rediagnosed is above the current average of the group it is entering (stage III). Adding it to the new set will, by definition, raise the average survival and thus improve the prognosis. In our study, there were 461 (79.1%) stage I/II patients in LNs < 12 group, but 747 (60.1%) stage I/II patients in LNs ≥ 12 group. The higher proportion of stage I/II patients in the LN < 12 group results suggest there is the possibility that stage migration has occurred due to insufficient harvested LNs in those patients.

There are several limitations and inherent selection bias to our study due to its retrospective nature. Although this study is the largest so far to examine the impact of LN involvement in patients with rectal cancer, the data may have been affected by unmeasured confounding variables, including different surgical techniques between surgeons, individual tumor biology, and varying tumor responses to adjuvant treatment. Nevertheless, the data that we present in this study hope to provide a baseline for further studies to verify the optimal number of harvested LNs in rectal cancer patients based on the presence or absence of pCRT.

This study suggests that the number of LNs harvested is an important prognostic factor in rectal cancer patients with pCRT. However, more importantly, it also suggested that the required number of harvested LNs varies according to treatment setting. The value of this study is in the analysis of patients based on treatment settings, and we have derived the minimum values of 12 LNs in the non pCRT group and 8 LNs in the pCRT group. We recommend that a different requirement for numbers of LN harvested should be used depending on the presence or absence of pCRT, and that this should be considered to be included in the next versions of staging systems for rectal cancer. Certainly, further successive studies are necessary to assess this further, before the optimal number of harvested LN is determined.

## 5 Conclusion

Our study demonstrates that the number of LNs harvested is an important prognostic factor in rectal cancer patients, and that based on presence or absence of pCRT, a different number of LNs may be optimal for staging and prognosis. Insufficient LN retrieval results in poorer prognosis in LN− patients, and this is likely due to stage migration. Examination of LNs ≥ 12 and LNs ≥ 8 should be aimed to enable accurate staging and optimal treatment for the non-pCRT group and pCRT group of patients, respectively.
